# GW501516-Mediated Targeting of Tetraspanin 15 Regulates ADAM10-Dependent N-Cadherin Cleavage in Invasive Bladder Cancer Cells

**DOI:** 10.3390/cells13080708

**Published:** 2024-04-19

**Authors:** Alexandre Barbaud, Isabelle Lascombe, Adeline Péchery, Sergen Arslan, François Kleinclauss, Sylvie Fauconnet

**Affiliations:** 1 SINERGIES–LabEx LipSTIC ANR-11-LABX-0021, Université de Franche-Comté, F-25000 Besançon, Franceisabelle.lascombe@univ-fcomte.fr (I.L.);; 2 CHU Besançon, Service Urologie, Andrologie et Transplantation Rénale, F-25000 Besançon, France; 3 CHU Besançon, Centre Investigation Clinique, Inserm CIC 1431, F-25000 Besançon, France

**Keywords:** bladder cancer, N-cadherin, ADAM10, tetraspanin 15, PPARβ/δ, GW501516

## Abstract

Bladder cancer aggressiveness is correlated with abnormal N-cadherin transmembrane glycoprotein expression. This protein is cleaved by the metalloprotease ADAM10 and the γ-secretase complex releasing a pro-angiogenic N-terminal fragment (NTF) and a proliferation-activating soluble C-terminal fragment (CTF2). Tetraspanin 15 (Tspan15) is identified as an ADAM10-interacting protein to induce selective N-cadherin cleavage. We first demonstrated, in invasive T24 bladder cancer cells, that N-cadherin was cleaved by ADAM10 generating NTF in the extracellular environment and leaving a membrane-anchored CTF1 fragment and that Tspan15 is required for ADAM10 to induce the selective N-cadherin cleavage. Targeting N-cadherin function in cancer is relevant to preventing tumor progression and metastases. For antitumor molecules to inhibit N-cadherin function, they should be complete and not cleaved. We first showed that the GW501516, an agonist of the nuclear receptor PPARβ/δ, decreased Tspan15 and prevented N-cadherin cleavage thus decreasing NTF. Interestingly, the drug did not modify ADAM10 expression, which was important because it could limit side effects since ADAM10 cleaves numerous substrates. By targeting Tspan15 to block ADAM10 activity on N-cadherin, GW501516 could prevent NTF pro-tumoral effects and be a promising molecule to treat bladder cancer. More interestingly, it could optimize the effects of the N-cadherin antagonists those such as ADH-1 that target the N-cadherin ectodomain.

## 1. Introduction

N-cadherin is a transmembrane glycoprotein mediating calcium-dependent intercellular adhesion [1]. It is expressed in neural, fibrous, and muscle tissues as well as in cardiomyocytes, fibroblasts, and glial and mesenchymal cells [2]. In addition to its adhesion function, N-cadherin is essential for several biological processes such as embryogenesis, cell migration, and cellular signaling [3]. In epithelial cancers, the abnormal expression of N-cadherin is associated with the loss of E-cadherin, a process named cadherin switch, which is a hallmark of the epithelial-mesenchymal transition reported in different cancers and in bladder cancer [4]. N-cadherin promotes tumor cell survival, migration, and invasion, and its high expression level is often associated with a poor prognosis [5]. In a previous study, we detected an aberrant expression of N-cadherin in high-grade non-muscle invasive bladder cancer confined to the lamina propria by immunohistochemistry and identified this adhesion molecule as an independent predictor of tumor progression [6].

Targeting N-cadherin function in cancer cells is relevant to preventing primary tumor progression and controlling the dissemination of metastases. This requires understanding the regulation of this adhesion molecule. N-cadherin is the target of a protease-mediated cleavage. In particular, ADAM10 (a disintegrin and metalloprotease 10) is responsible for an initial proteolytic processing of N-cadherin leading to the release of a N-terminal extracellular fragment NTF (95 kDa) and leaving a membrane-anchored C-terminal fragment CTF1 (40 kDa) at the cytoplasmic surface. CTF1 is further cleaved by the presenilin-1/γ-secretase complex producing a soluble CTF2 (35 kDa) [7,8]. NTF and CTF2 fragments are implicated in different cellular signaling pathways. CTF2 acts as a transcription regulator promoting β-catenin-mediated target gene expression, such as *cyclin D1* proliferating gene [9], and repressing CBP/CREB-mediated transcription by triggering CBP proteasomal degradation [7]. NTF stimulates neurite outgrowth through the fibroblast growth factor receptor-dependent activation [10]. It increases endothelial cell migration and angiogenesis [11], as well as the survival of endothelial cells and vascular smooth muscle cells [12]. In patients with malignant bone and soft tissue tumors, a high level of serum N-cadherin/NTF was correlated with a high histological grade and tumor size (>5 cm). In addition, it was associated with a lower rate of disease-free survival, local recurrence-free survival, metastasis-free survival, and overall survival [13]. More elevated concentrations of soluble N-cadherin were detected in the serum of prostate cancer patients compared to their healthy counterparts [14].

In the fight against cancer, blocking NTF generation by targeting ADAM10 proteolytic activity could be a relevant therapeutic strategy. In fact, ADAM10 is highly upregulated in cancer, including bladder cancer [15]. Preventing its expression or activity is therefore being considered as an anti-cancer therapy. However, ADAM10 is responsible for the extracellular region shedding of a large variety of transmembrane proteins such as Notch, amyloid precursor protein, prion protein, growth factor/cytokine receptors, adhesion molecules, and tumor antigens. Through the cleavage of some of these cell surface substrates, ADAM10 is implicated in development and neuroprotection [16]. Direct action on ADAM10 activity could result in multiple toxic side effects. It is therefore important to avoid blocking its proteolytic activity.

Recently, the transmembrane protein tetraspanin 15 (Tspan15) has been characterized as a positive regulator of ADAM10-promoted cleavage of N-cadherin [17,18,19]. Tspan15 belongs to a family of six tetraspanins (Tspan5, Tspan10, Tspan14, Tspan15, Tspan17, and Tspan33) that have been identified as specific ADAM10-interacting protein partners. They form the TspanC8 subgroup characterized by the presence of eight cysteines in the large extracellular loop [20]. According to TspanC8 associated with ADAM10, the enzyme has specificity for a given substrate. Specifically targeting Tspan15 seems more appropriate to block the enzyme activity of ADAM10 on N-cadherin.

Our aim was to prevent the proteolytic activity of the molecular scissor formed by the Tspan15/ADAM10 complex using a pharmacological inhibitory strategy to block the production of N-cadherin/NTF. Thus, we evaluated the inhibitory capacity of the GW501516 drug (IUPAC: {4-[({4-methyl-2-[4-(trifluoromethyl)phenyl]-1,3-thiazol-5-yl}methyl)sulfanyl]-2-methylphenoxy}acetic acid) on the function of the Tspan15/ADAM10 complex. GW501516 is a high-affinity synthesized agonist of the nuclear receptor PPARβ/δ (Peroxisome Proliferator-Activated Receptor β/δ) [21]. In a previous study, we reported that exposure of proliferating T24 invasive bladder cancer cells to 25 µM GW501516 led to a diminution in cell viability, a G2/M cell cycle arrest, and the triggering of apoptosis [22]. In addition, in same cells, 15 µM drug decrease the full-length N-cadherin level [23].

In this study, we demonstrated (1), for the first time, N-cadherin shedding in invasive urothelial cancer cells by ADAM10, (2) the involvement of Tspan15 in ADAM10-mediated cleavage of N-cadherin, (3) the requirement of Tspan15 inhibition to decrease NTF, and (4), for the first time, in addition to reducing the full-length N-cadherin, the inhibitory effect of GW501516 on N-cadherin/NTF releasing through the decrease of Tspan15 level and consequently on ADAM10 proteolytic activity.

## 2. Materials and Methods

### 2.1. Chemicals

The PPARβ/δ agonist GW501516 was provided by Enzo Life Sciences (Villeurbanne, France). L-165041 (another PPARβ/δ agonist) was obtained from Calbiochem (San Diego, CA, USA). Batimastat (broad spectrum matrix metalloprotease inhibitor), GI254023X (selective ADAM10 metalloproteinase inhibitor), and DAPT (selective γ-secretase inhibitor) were obtained from R&D Systems (Lille, France). GSK0660 (PPARβ/δ antagonist) was obtained from CliniSciences (Nanterre, France). The final concentration of organic solvent (dimethylsulfoxide) was less than 0.1%, which had no effect on cell viability.

### 2.2. Cell Culture and Treatments

The T24 human bladder cancer cell line was obtained from ATCC (ATCC^®^HTB-4^TM^). T24 cells were maintained in Mc COY’s 5a medium containing L-glutamine and supplemented with 10% fetal calf serum (FCS) (Invitrogen, Cergy Pontoise, France), 1% antibiotic antimycotic mixture (10 mg/mL streptomycin, 10,000 units/mL penicillin, 25 µg/mL amphotericin B), and 15 mM Hepes (Sigma, Saint-Quentin-Fallavier, France) at 37 °C in a humidified 5% CO_2_, 95% O_2_ air incubator. Cells were tested for the absence of mycoplasma before the beginning of experiments. According to the experiments, cells were seeded in 12- or 6-well plates and cultured with 5% FCS. Before any treatment, cells were washed twice with FCS-free medium and then incubated with 10 µM DAPT, 5 µM Batimastat, GI254023X, or different concentrations of GW501516 (1, 15, or 25 µM) with or without the PPARβ/δ antagonist GW0660 (10 µM) or L-165041 (15 µM). Drug exposure times vary according to the experiments.

### 2.3. Western Blotting Analysis

After treatment, cells were washed with PBS 1X and scraped in lysis buffer RIPA (50 mM Tris-HCl pH 7.4, 150 mM NaCl, 1 mM EDTA, 1% Nonidet P40, 0.5% sodium desoxycholate) supplemented with protease inhibitors (Roche Diagnostics, Meylan, France). Then, whole cell lysates were sonicated and centrifuged at 10,000× *g* for 10 min at 4 °C. Protein concentration was estimated using the Bio-Rad protein assay (Bio-Rad, Marnes-la-Coquette, France). Total protein extracts (30 µg) were solved in Laemmli buffer (Bio-Rad) and separated by a 7.5 or 12% SDS-PAGE. Proteins were transferred onto PVDF membranes (GE Healthcare, Buckinghamshire, UK), and non-specific binding was blocked in TBS-Tween 20 buffer (0.5 mM Tris-HCl, 45 mM NaCl, 0.05% Tween 20, pH 7.4) containing 5% nonfat milk or bovine serum albumin. Membranes were incubated with the following appropriate primary antibodies: Anti-ADAM10 (clone 14194, 1:1000) was from Cell Signaling (Ozyme, St Quentin en Yvelines, France). Anti-β-actin (clone AC-15, 1:40,000) and anti-N-cadherin (clone GC-4, 1:1000) were from Sigma. Anti-PPARβ/δ (clone H-74, 1/500) was from Santa Cruz Biotechnology (Clinisciences, Nanterre, France). Anti-N-cadherin (clone 3B9, 1/2000) and anti-tetraspanin 15 (#PA5-42948, 1/1000) were from ThermoFisher Scientific (Illkirch, France). Bound primary antibodies were detected using HRP-conjugated secondary antibodies: anti-rabbit IgG (1:5000 or 1:10,000), anti-mouse IgG (1:10,000), and anti-goat IgG (1/10,000) were from Dako Agilent Technologies (Les Ulis, France). Proteins were visualized by using an enhanced chemiluminescence detection method (GE Healthcare) followed by film exposure (Hyperfilm ECL, GE Healthcare) or by using ChemiDoc XRS+ (version 5.1) with image lab software (Bio-Rad). Densitometric analysis was performed using both Image J software (version 1.54i) and ChemiDoc XRS+ with image lab software (version 1709690).

### 2.4. Acetone Precipitation of T24 Cell Culture Supernatants

T24-cell conditioned media were collected and centrifuged (300× *g*, 10 min, room temperature; 2000× *g*, 10 min, room temperature) to discard all cellular fragments (living and dead cells). Supernatants were then concentrated using Pierce^TM^ Protein concentrators PES 3K (Thermo Fisher Scientific, Illkirch, France). Concentrated supernatants were diluted in 4 volumes of cold (−20 °C) acetone and precipitated samples were stored overnight at −80 °C. The precipitates were centrifuged (10,000× *g*, 10 min, 4 °C) and the supernatants were carefully removed. Pellets were air-dried at room temperature to eliminate any acetone residue. For SDS-PAGE analysis, pellets were resuspended in 2X Laemmli buffer.

### 2.5. RNA Isolation and RTq-PCR Analysis

Total RNA was extracted using TRI REAGENT^®^ (Euromedex, Mundelsheim, France). Contaminating genomic DNA was removed with an RNase-free DNase I treatment (Invitrogen) according to the manufacturer’s instructions. Total RNA (3 µg) was then reverse transcribed into cDNA with 200 U MMLV RT (Invitrogen) and 500 ng oligo(dT) primers (Invitrogen) following the manufacturer’s guidelines. PCR assays were performed with the 7500 Real Time PCR System (Applied Biosystems, Courtaboeuf, France) using TaqMan or SYBR^®^Green technology. PCR using TaqMan technology was performed in a final volume of 20 μL containing 12.5 μL of TaqMan Gene Expression PCR Master Mix (Applied Biosystems), 5 µL of cDNA diluted 1:20, 100 nM of *cdh2* or *atp5β* TaqMan probe (Eurogentec, Seraing, Belgium), and 1 µM of each primer (Eurogentec) for *cdh2* and 500 nM for *atp5β*. PCR with SYBR^®^ Green technology was performed in a reaction mixture of 20 µL containing 12.5 µL of Fast Start DNA Master Hybridization probe (Applied Biosystems), 5 µL of cDNA diluted 1:20, 100 nM of each primer for *Adam10*, 200 nM for *Tetraspanin,* and *rpl38* or 300 nM for *atp5β* and *Adrp*. Primer and probe sequences are listed in Table S1. The Taq polymerase was activated at 50 °C for 2 min, followed by a denaturation step at 95 °C for 10 min. Then, whatever technology was used, PCR mixtures were subjected to 40 cycles of amplification. The following PCR cycle settings were used: denaturation at 95 °C for 15 sec, hybridization/elongation at 60 °C for 1 min. Each reaction was run in triplicates with three independent experiments. The fold difference in expression of the target genes between control and treated cells was calculated by the 2^−∆∆Ct^ method [24]. *ATP5β* and *rpl38* were used as reference genes.

### 2.6. RNA Interference and Cell Transfection

Control siRNA (negative control for evaluating RNAi off-target effects, sc-37007), *PPARβ/δ* (sc-36305), *Adam10* (sc-41410), and *Tspan15* (sc-90664) specific siRNA (pool of 3 target-specific 19–25 nucleotide siRNAs) were from Santa Cruz Biotechnology. T24 cells were seeded in 24-well plates (80,000 cells/well) and cultured in Mc COY’s 5a medium with 5% FCS without antibiotics. After 24 h, at 70–80% confluence, cells were transfected with 10, 25, or 50 nM siRNA using Lipofectamine^TM^ 2000 reagent (Invitrogen) according to the manufacturer’s instructions. After 24 h transfection, cells were incubated in serum-free medium without (control cells) or with 15 µM GW501516 for 24 h more and then were harvested for RNA or protein analyses.

### 2.7. Plasmids and Transfection

Control plasmid and plasmid encoding Tspan15 protein were purchased from VectorBuilder (Chicago, IL, USA). T24 cells were seeded in 24-well plates and cultured in Mc COY’s 5a medium with 5% FCS without antibiotics. At 85–90% confluency, cells were transfected with increasing concentrations of plasmid (250, 500, 1000 ng) using Lipofectamine^TM^ 2000 reagent (Invitrogen) as specified by the manufacturer’s recommendations. After 24 h transfection, cells were incubated in a serum-free medium for 24 h more and then were harvested for RNA or protein analyses.

### 2.8. Statistical Analysis

Data were expressed as mean ± SD of three independent experiments or as specified for each figure. The significant differences between groups were evaluated using the two-tailed unpaired Student’s *t*-test. A *p*-value < 0.05 was considered statistically significant.

## 3. Results

### 3.1. N-Cadherin Cleavage over Time in T24 Bladder Carcinoma Cells

**Figure 1:**
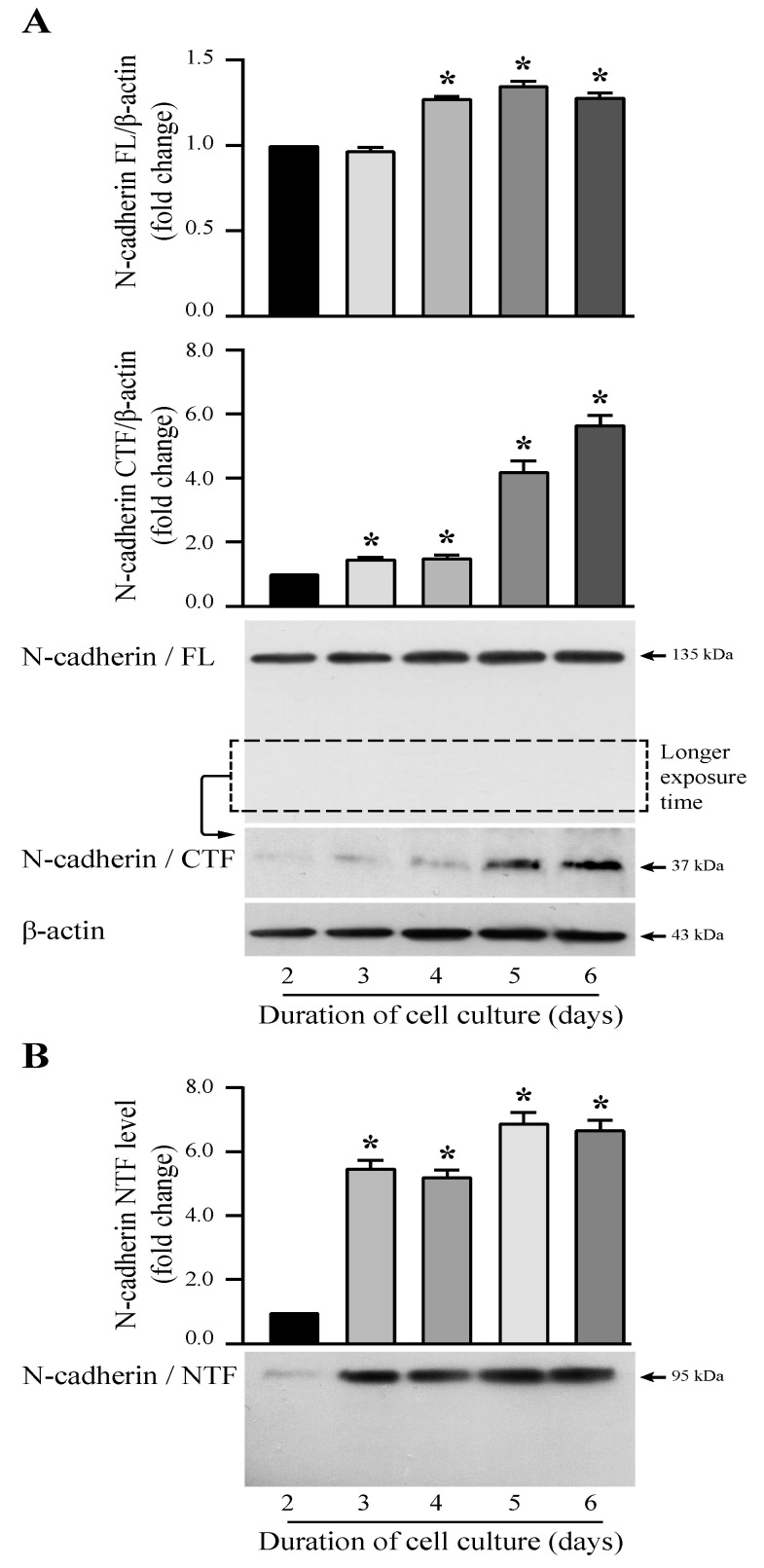
Detection of N-cadherin cleavage fragments according to T24 cell density. Cells were seeded in a medium containing 5% serum for 2, 3, 4, 5, or 6 days. At each experimental time, they were incubated in a serum-free medium for the last 24 h. (**A**) A C-terminal fragment (CTF) was revealed by Western-blotting analysis from whole cell lysates with the 3B9 antibody directed against the intracellular part of N-cadherin. β-actin was used as an internal loading control. (**B**) An N-terminal fragment (NTF) was detected in T24 cell-conditioned media with the GC-4 antibody directed against the extracellular domain of N-cadherin. The graphs depict densitometric analysis results of Western blots by using ImageJ. Data are means ± SD of three independent experiments performed in triplicates (* *p* < 0.05).

To assess the cell density from which N-cadherin cleavage was observed in invasive bladder cancer cells, T24 cells were seeded in a 5% FCS-supplemented medium and were cultured for 2, 3, 4, 5, and 6 days. For each time point, cells were incubated in a serum-free medium for 24 h, and the experiment was stopped to harvest proteins for analysis. The expression of the 135 kDa entire N-cadherin increased very slightly with the cell density (Figure 1A). At 3 days of culture, a 37 kDa C-terminal fragment (CTF) was slightly detected with a maximum from 5 days of culture (Figure 1A) as well as a 95 kDa N-terminal fragment (NTF) in cell-supernatants (Figure 1B). It should be noted that we had to expose the autoradiographic film for a longer time to observe the CTF fragment, which is probably CTF1. These results suggested that the presenilin-1/γ-secretase complex is inhibited in confluent T24 cells. The presence of these fragments meant that N-cadherin was cleaved in untreated T24 cells corresponding to controls. For all following experiments, cells were cultured for 5 days before analysis. Specifically, they were incubated in a 5% FCS-supplemented medium for 4 days, and after two washes in a serum-free medium, they were placed in FCS-free medium one day more (thus 5 days in culture), with or without treatment in the presence of a chemical molecule. We stopped our experiments after 5 days of culture (4 days in 5% FCS and 24 h in FSC-free medium) because, at this experimental time point, we had an increase of the N-cadherin full-length and a meaningful rise of CTF and NTF levels.

### 3.2. ADAM10 and γ-Secretase Complex Induced N-Cadherin Cleavage

**Figure 2:**
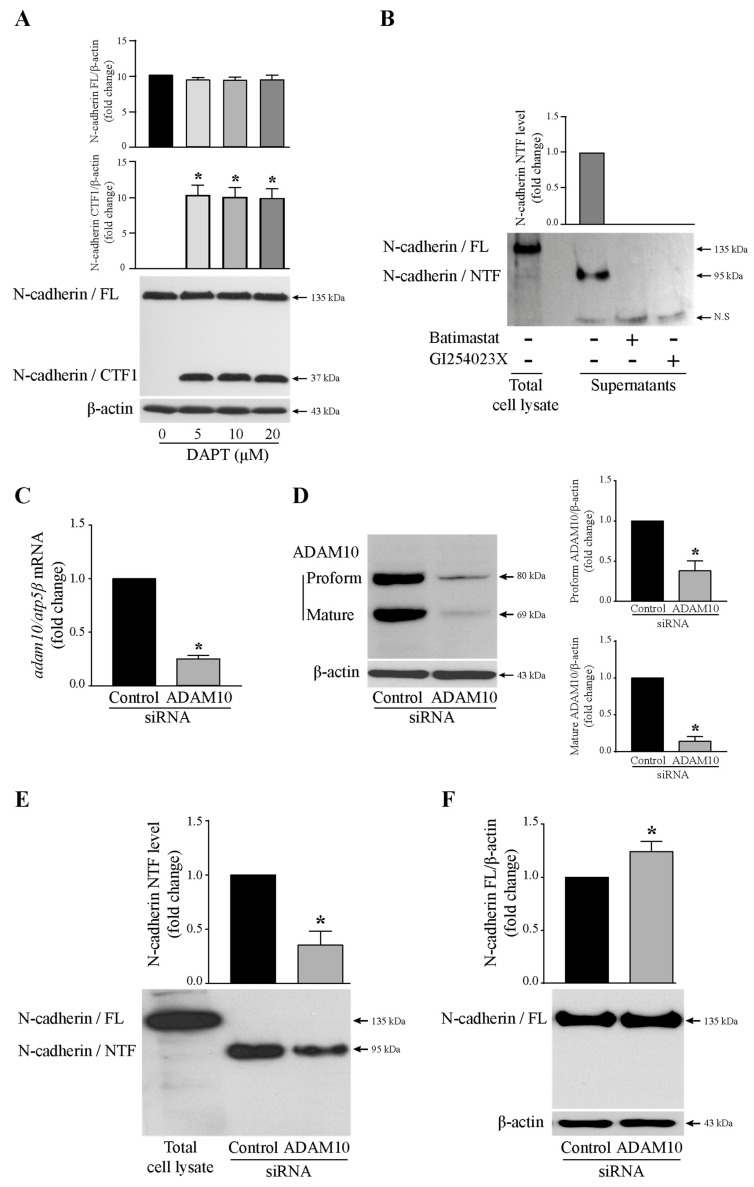
Involvement of ADAM10 and γ-secretase complex in N-cadherin cleavage. (**A**) T24 cells were treated with increasing concentrations of the γ-secretase inhibitor DAPT (5, 10, 20 µM) for 24 h. Total cell lysates were subjected to immunoblotting with the 3B9 antibody raised against the cytoplasmic domain to reveal the membrane-anchored CTF1 of N-cadherin. β-actin was used as an internal loading control. The graphs depict densitometric analysis results of Western blots by using ImageJ. Data are means ± SD of three independent experiments performed in triplicates (* *p* < 0.05). (**B**) Cells were treated with 5 µM Batimastat (a broad-spectrum matrix metalloprotease inhibitor) or 5 µM GI254023X (a selective ADAM10 metalloproteinase inhibitor) for 24 h. The T24 cell culture supernatants were analyzed by Western blotting with GC-4 antibody to detect the N-cadherin extracellular domain (NTF). Total cell lysate was used as a positive control for N-cadherin expression. The graph depicts densitometric analysis results of Western blots by using ImageJ. Data are means ± SD of three independent experiments performed in triplicates (* *p* < 0.05). (**C**) Validation of ADAM10 knockdown efficiency at the mRNA level by RTq-PCR analysis in T24 cells transfected with 25 nM ADAM10 siRNA. (**D**) Western blotting analysis of ADAM10 protein (proform and mature form) depletion in ADAM10 siRNA transfected T24 cells. β-actin was used as an internal loading control. The graphs depict densitometric analysis results of Western blots by using ImageJ. Data are means ± SD of three independent experiments performed in triplicates (* *p* < 0.05). (**E**) N-cadherin/NTF level detection in T24 cell-supernatants from ADAM10 siRNA transfected cells by Western blotting. Total cell lysate was used as a positive control for N-cadherin expression. The graph depicts densitometric analysis results of Western blots by using ImageJ. Data are means ± SD of three independent experiments performed in triplicates (* *p* < 0.05). (**F**) N-cadherin full-length expression analysis by Western blotting in ADAM10 siRNA transfected cells. β-actin was used as an internal loading control. The graph depicts densitometric analysis results of Western blots by using ImageJ. Data are means ± SD of three independent experiments performed in triplicates (* *p* < 0.05).

To specify the proteases involved in N-cadherin cleavage, T24 cells were treated with specific chemical inhibitors to prevent the activity of the enzymes. DAPT was used to inhibit the γ-secretase complex and thus to avoid CTF1 cleavage. Batimastat (a broad-spectrum matrix metalloprotease inhibitor) or GI254023X (a selective ADAM10 metalloproteinase inhibitor) were used to suppress ADAM10 activity and thus NTF generation. As shown in Figure 2A, cells were incubated with increasing concentrations of DAPT (5, 10, 20 µM). From whole cell lysates, N-cadherin full-length was not modified. CTF1 fragment was observed at 5 µM DAPT with a maximum level indicating that this inhibitor concentration was sufficient to block the enzyme activity. The remaining CTF1 fragment was not cleaved into CTF2 in the presence of the γ-secretase inhibitor, which is why it was still disclosed on immunoblot. Therefore, in Figure 1, the CTF fragment observed at the size of 37 kDa was CTF1. As presented in Figure 2B, cells were stimulated or not with 5 µM Batimastat or GI254023X for 24 h. The GC-4 antibody directed against the extracellular domain of N-cadherin was used. Thus, it can detect both the entire form of N-cadherin in whole cell lysates and the N-cadherin/NTF in the extracellular medium. Soluble N-cadherin/NTF level was determined from acetone-precipitated T24 cell culture supernatants with this antibody. NTF was detected in untreated T24 cell-conditioned media but, as expected, was no longer found in MMP/ADAM10 inhibitor-exposed cells, indicating that the enzyme was completely inhibited. From there, we focused our study on ADAM10-mediated ectodomain shedding of N-cadherin. Thus, to confirm the involvement of this enzyme in the generation of N-cadherin/NTF, cells were transiently transfected with siRNA targeting ADAM10 to knockdown ADAM10 protein expression, or a scrambled siRNA used as a negative control. As expected, analyses of ADAM10 mRNA by RTq-PCR or ADAM10 protein by Western blotting validated the decreased expression of the enzyme in T24 cells. Thus, in ADAM10 siRNA-transfected cells, and in comparison with control siRNA-transfected cells, a significant depletion of ADAM10 was observed at both mRNA (Figure 2C) and protein levels for both pro- and mature forms (Figure 2D). As a result, a significant decrease in the amount of NTF released in T24 cell-supernatants was observed (Figure 2E). As expected, this was associated with an increase in full-length N-cadherin (Figure 2F). Thus, for the first time in T24 bladder cancer cells, our results showed that the full-length N-cadherin was a substrate for ADAM10. Through cleavage of this membrane protein, the enzyme releases the NTF fragment into the extracellular environment. When it is inhibited, NTF is no longer detected.

### 3.3. Tspan15 Involvement in N-Cadherin Extracellular Cleavage

**Figure 3:**
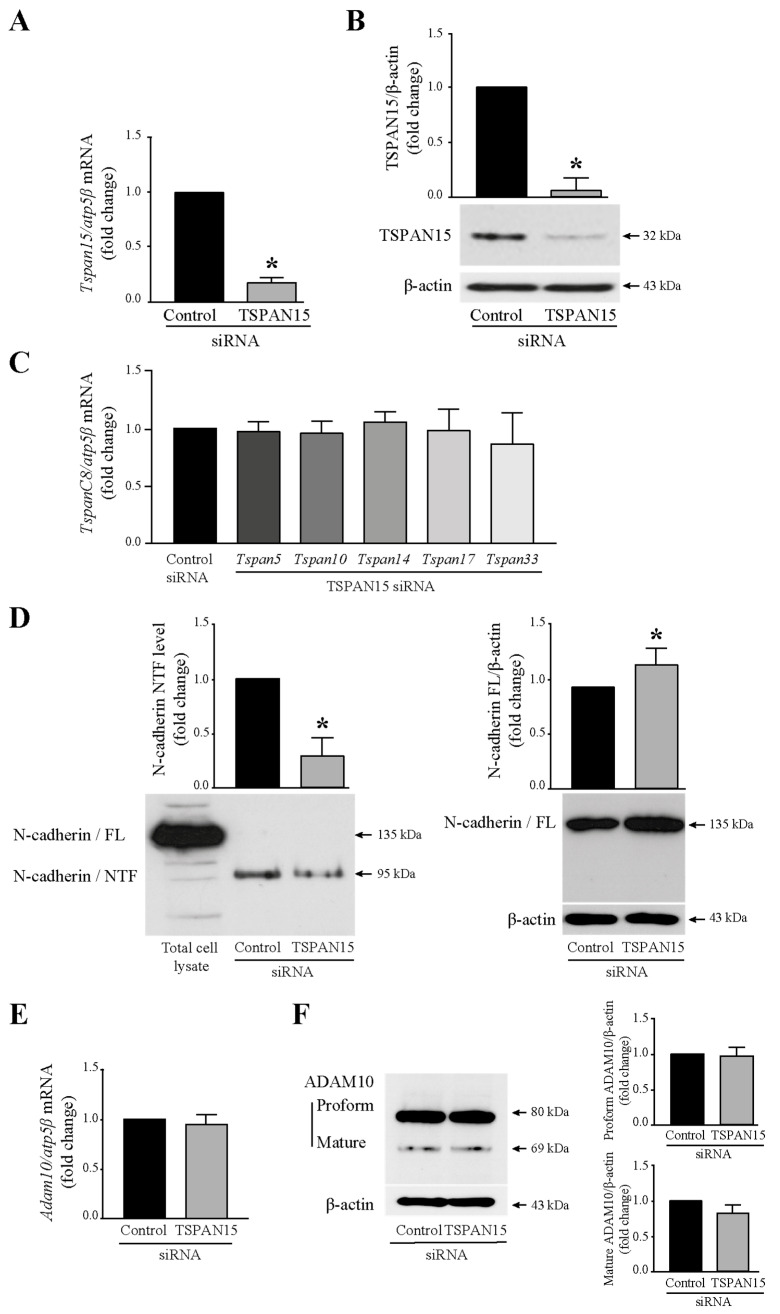
Tspan15 controls ADAM10-mediated cleavage of N-cadherin in T24 cells. (**A**) Validation of Tspan15 knockdown efficiency at the mRNA level by RTq-PCR analysis in T24 cells transfected with 25 nM TSPAN15 siRNA. Data are means ± SD of three independent experiments performed in triplicates (* *p* < 0.05). (**B**) Western blotting analysis of TSPAN15 protein depletion in TSPAN15 siRNA transfected T24 cells. β-actin was used as an internal loading control. The graph depicts densitometric analysis results of Western blots by using ImageJ. Data are means ± SD of three independent experiments performed in triplicates (* *p* < 0.05). (**C**) Expression analysis of the different members of the TspanC8 group in TSPAN15 siRNA transfected T24 cells. (**D**) N-cadherin/NTF level detection in T24 cell-supernatants from TSPAN15 siRNA transfected cells by Western blotting. Total cell lysate was used as a positive control for N-cadherin expression. The graph depicts densitometric analysis results of Western blots by using ImageJ. Data are means ± SD of three independent experiments performed in triplicates (* *p* < 0.05). N-cadherin full-length expression analysis by Western blotting in TSPAN15 siRNA transfected cells. β-actin was used as an internal loading control. The graph depicts densitometric analysis results of Western blots by using ImageJ. Data are means ± SD of three independent experiments performed in triplicates (* *p* < 0.05). (**E**) *Adam10* mRNA expression analysis by RTq-PCR in T24 cells transfected with 25 nM TSPAN15 siRNA. Data are means ± SD of three independent experiments performed in triplicates. (**F**) Western blotting analysis of ADAM10 protein expression (proform and mature form) in TSPAN15 siRNA transfected T24 cells. β-actin was used as an internal loading control. The graphs depict densitometric analysis results of Western blots by using ImageJ. Data are means ± SD of three independent experiments performed in triplicates.

To examine whether Tspan15, described as a regulator of the selective cleavage of ADAM10 for N-cadherin [17] is involved in N-cadherin ectodomain shedding observed in T24 cells, its expression was analyzed by silencing its transcripts. Thus, T24 cells were transiently transfected with siRNA targeting Tspan15 to knockdown Tspan15 protein expression, or a scrambled siRNA was used as a negative control. As expected, Tspan15 knockdown by 25 nM Tspan15 siRNA, resulted in the reduction of Tspan15 mRNA level and consequently of Tspan15 protein level (Figure 3A,B). This Tspan15 siRNA was specific since the transcript expression of the other members of the TspanC8 family (Tspan5, Tspan10, Tspan14, Tspan17, and Tspan33) was not abolished (Figure 3C) in Tspan15 siRNA transfected T24 cells compared to cells transfected with control siRNA. A significant decrease of Tspan15 following the Tspan15 siRNA transfection was associated with the diminution of N-cadherin/NTF production and the increased expression of the full-length N-cadherin (Figure 3D). It is worth noting that the Tspan15 silencing did not impact the expression of ADAM10 at both mRNA (Figure 3E) and proform and mature protein levels (Figure 3F). Taken together, these data indicated, for the first time in T24 bladder cancer cells, that Tspan15 was implicated in the ectodomain cleavage of N-cadherin by ADAM10 since blocking Tspan15 alone reduced the NTF amount released in the extracellular compartment.

### 3.4. Overexpression of Tspan15 Increased N-Cadherin/NTF Generation

**Figure 4:**
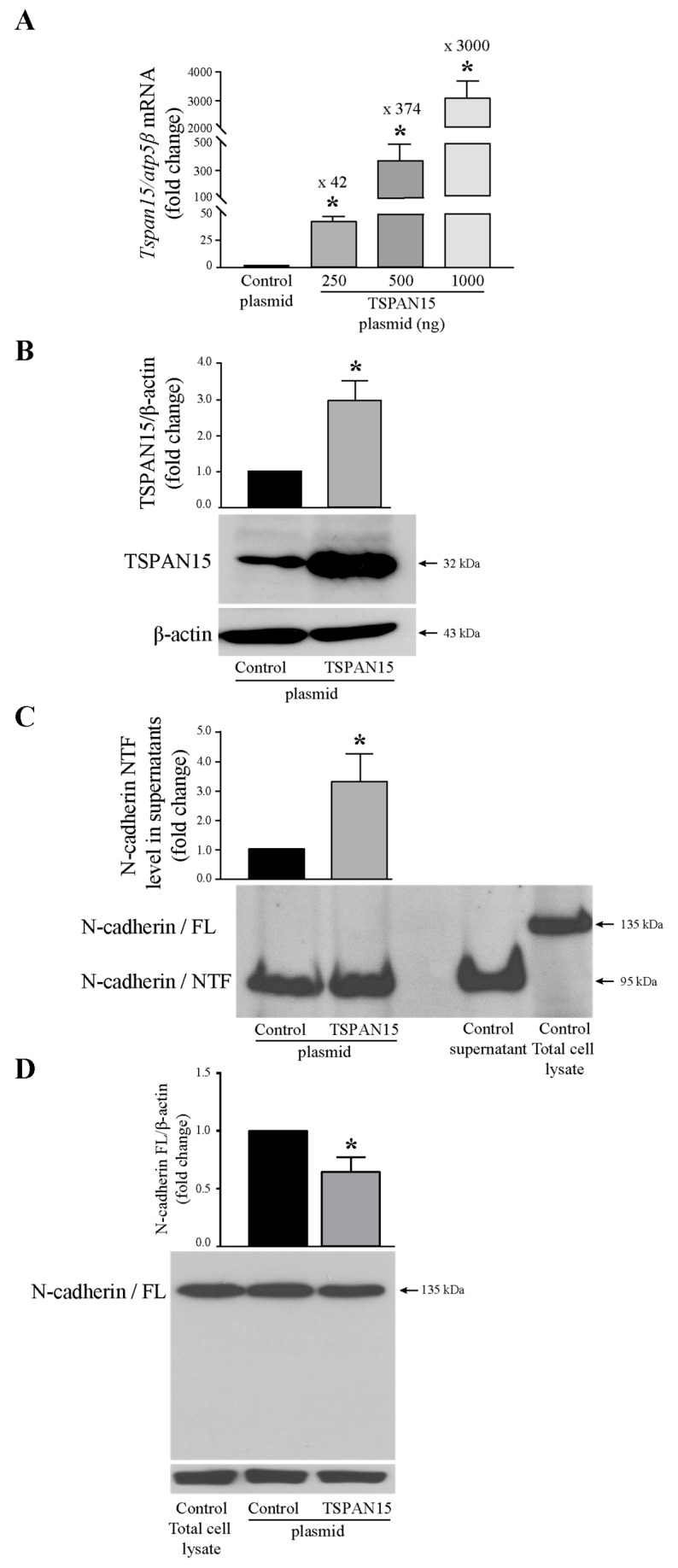
N-cadherin/NTF is raised upon increasing amounts of Tspan15. (**A**) *Tspan15* mRNA expression analysis by RTq-PCR in T24 cells after transfection of empty vector (control plasmid) or increasing amounts of TSPAN15 plasmid (250, 500, 1000 ng). Fold inductions represent a comparison with control plasmid transfected cells (set at 1). (**B**) Western blotting analysis of TSPAN15 protein expression in TSPAN15 plasmid transfected T24 cells. β-actin was used as an internal loading control. The graph depicts densitometric analysis results of Western blots by using ImageJ. Data are means ± SD of three independent experiments performed in triplicates (* *p* < 0.05). (**C**) N-cadherin/NTF level detection in T24 cell-supernatants from TSPAN15 plasmid transfected cells by Western blotting. Control supernatant was used as a positive control for NTF production in non-transfected cells, and total cell lysate was used as a positive control for N-cadherin expression. The graph depicts densitometric analysis results of Western blots by using ImageJ. Data are means ± SD of three independent experiments performed in triplicates (* *p* < 0.05). (**D**) N-cadherin full-length expression analysis by Western blotting in TSPAN15 plasmid transfected cells. Control total cell lysate was used as a positive control for N-cadherin expression. β-actin was used as an internal loading control. The graph depicts densitometric analysis results of Western blots by using ImageJ. Data are means ± SD of three independent experiments performed in triplicates (* *p* < 0.05).

To confirm the involvement of Tspan15 in N-cadherin extracellular cleavage, we evaluated the level of N-cadherin/NTF after Tspan15 protein overexpression experiments carried out by transfecting T24 cells with a vector encoding Tspan15 protein. Increasing amounts of Tspan15 plasmid were transfected in T24 cells, and mRNA and protein expression were evaluated by RTq-PCR and Western blotting analyses. As shown in Figure 4A, Tspan15 mRNA level increased 42-, 374-, and 3000-fold with 250, 500, and 1000 ng Tspan15 construct, respectively, indicating that the transfection was effective. The Tspan15 transcript overexpression obtained in T24 cells transfected with 250 ng Tspan15 plasmid was associated with an increase in the Tspan15 protein level (Figure 4B). As expected, and compared with control plasmid-transfected cells, the level of N-cadherin/NTF in T24 supernatants was higher in Tspan15 plasmid-transfected cells (Figure 4C), and this was associated with a decrease of the full-length N-cadherin (Figure 4D). Control supernatant and total cell lysate from non-transfected cells were used as positive controls for GC-4 antibody binding. These data showed that an increase of Tspan15 expression in cells leads to a rise of NTF amount in the extracellular medium, meaning that ADAM10 proteolytic activity is enhanced.

### 3.5. GW501516 Exposure Reduced N-Cadherin Extracellular Fragment Releasing

**Figure 5:**
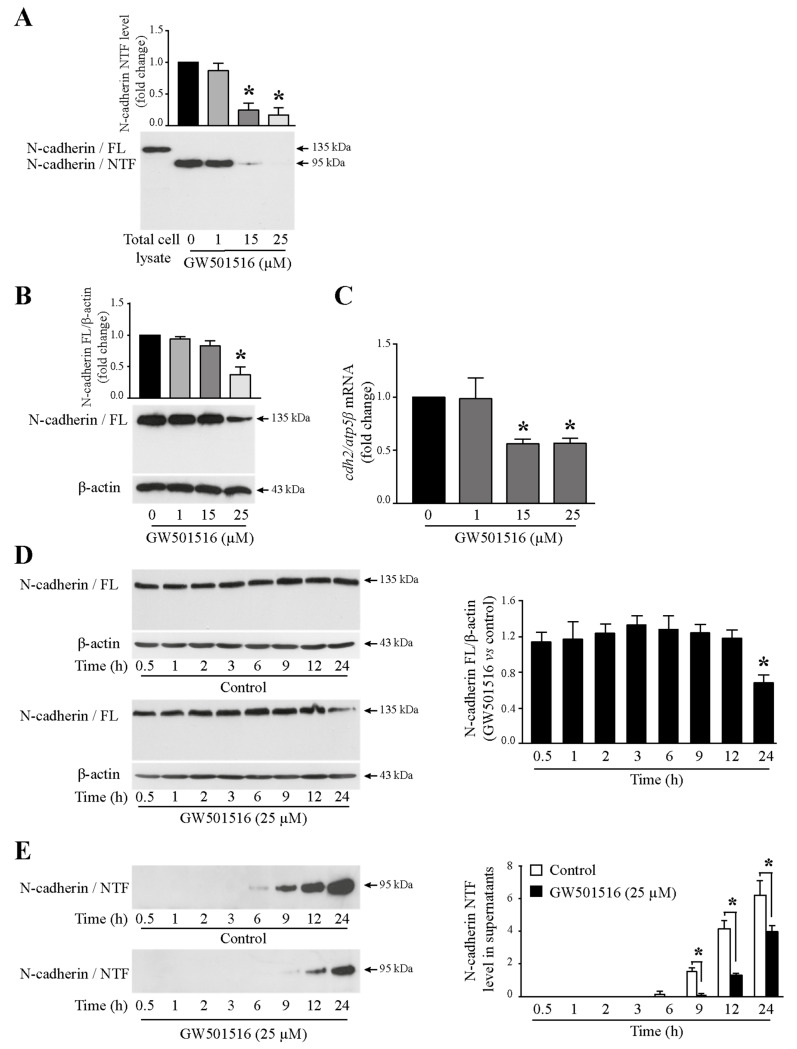
Reduction of N-cadherin/NTF release after GW501516 treatment of T24 cells. (**A**) T24 cells were treated with increasing concentrations of GW501516 (1, 15, 25 µM). The culture supernatants were analyzed by Western blotting with GC-4 antibody to detect the N-cadherin extracellular domain (NTF). Total cell lysate was used as a positive control for N-cadherin expression. The graph depicts densitometric analysis results of Western blots by using ImageJ. Data are means ± SD of three independent experiments performed in triplicates (* *p* < 0.05). (**B**) N-cadherin full-length expression analysis by Western blotting in T24 cells stimulated with increasing concentrations of GW501516 (1, 15, 25 µM). β-actin was used as an internal loading control. The graph depicts densitometric analysis results of Western blots by using ImageJ. Data are means ± SD of three independent experiments performed in triplicates (* *p* < 0.05). (**C**) *Cdh2* mRNA expression was analyzed by RTq-PCR. Fold inductions represent a comparison with vehicle-treated cells (set at 1) in the absence of GW501516. Data are means ± SD of three independent experiments performed in triplicates (* *p* < 0.05). (**D**) Kinetic of GW501516 effect on N-cadherin full length. T24 cells were treated or not with 25 µM GW501516 for the indicated times. N-cadherin full-length expression was analyzed by Western blotting. β-actin was used as an internal loading control. The graph depicts densitometric analysis results of Western blots by using ImageJ. Fold inductions represent a comparison with vehicle-treated cells (set at 1) in the absence of GW501516 for each experimental time point. Data are means ± SD of three independent experiments performed in triplicates (* *p* < 0.05). (**E**) Kinetic of GW501516 effect on NTF generation. T24 cells were treated or not with 25 µM GW501516 for the indicated times. The culture supernatants were analyzed by Western blotting with GC-4 antibody. The graph depicts densitometric analysis results of Western blots by using ImageJ. Data are means ± SD of three independent experiments performed in triplicates (* *p* < 0.05).

N-cadherin/NTF was reported to promote angiogenesis [11], which is an essential process for tumor growth and metastasis development [25]. Our objective was to block NTF production through the inhibition of N-cadherin extracellular cleavage by using GW501516, a PPARβ/δ chemical agonist. In a previous work [22], we showed that this molecule reduced the viability of T24 cells, and this result is confirmed in this study (Figure S2), demonstrating the anti-cancer effect of GW501516 on the bladder. To evaluate the effect of the drug on NTF production, T24 cells were treated or not with increasing concentrations of GW501516 (0, 1, 15, 25 µM) for 24 h. Soluble N-cadherin/NTF level was analyzed from acetone-precipitated cell culture supernatants by Western blotting. As shown in Figure 5A, the NTF generation was reduced from 15 µM GW501516 with a greater effect at 25 µM. Unexpectedly, we did not observe an increase of the full-length N-cadherin expression from the whole cell lysates but rather a significant decrease from the 15 µM drug (Figure 5B). This was associated with a decrease of *cdh2* transcripts at the same concentrations (Figure 5C). This result confirms what we had already observed [23], meaning that GW501516 decreases the entire form of N-cadherin. Therefore, the agonist reduces both full-length N-cadherin and the extracellular fragment NTF.

A kinetic of GW501516 effect was then performed with cells treated or not with the PPARβ/δ agonist (25 µM) for 0.5, 1, 2, 3, 6, 9, 12, and 24 h. A significant decrease of the full-length N-cadherin was observed after 24 h treatment (Figure 5D). NTF was faintly detected from 6 h with a maximum rate at 24 h in control cells. Interestingly, soluble N-cadherin was no longer spotted at 6 and 9 h in GW501516-stimulated cells and remained detected at 12 and 24 h but to a lesser extent in comparison with the control condition (Figure 5E). Overall, these results allowed us to identify a drug (GW501516) able to decrease the generation of N-cadherin/NTF in invasive bladder cancer cells.

### 3.6. GW501516 Did Not Impact the Expression of ADAM10 in T24 Cells

**Figure 6:**
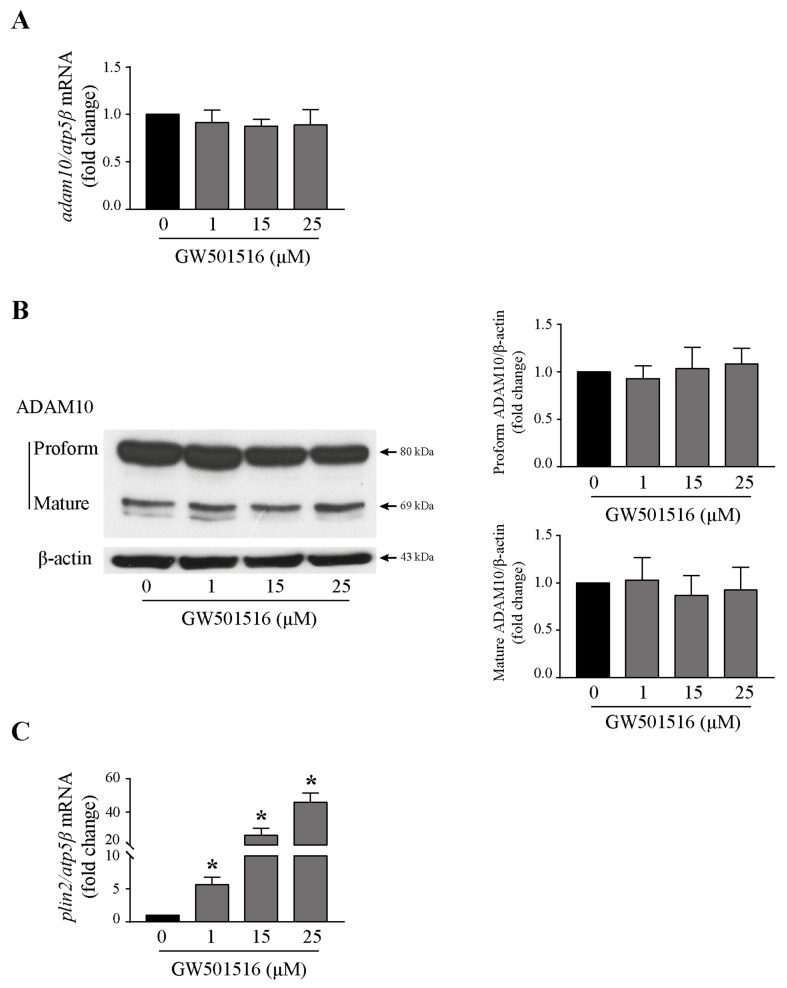
ADAM10 is not regulated by PPARβ/δ in T24 cells. Cells were treated with increasing concentrations of GW501516 (1, 15, 25 µM) for 24 h. (**A**) *Adam10* mRNA expression was analyzed by RTq-PCR. Fold inductions represent a comparison with vehicle-treated cells (set at 1) in the absence of GW501516. Data are means ± SD of three independent experiments performed in triplicates. (**B**) Western blotting analysis of ADAM10 protein (proform and mature form) in control and stimulated cells. β-actin was used as an internal loading control. The graphs depict densitometric analysis results of Western blots by using ImageJ. Data are means ± SD of three independent experiments performed in triplicates. (**C**) *Plin2*, a PPARβ target gene, was used as a positive control to validate the efficiency of GW501516. *Plin2* mRNA expression was analyzed by RTq-PCR. Fold inductions represent a comparison with vehicle-treated cells (set at 1) in the absence of GW501516. Data are means ± SD of three independent experiments performed in triplicates (* *p* < 0.05).

The association of GW501516-mediated decrease of NTF production with a diminution of ADAM10 proteolytic action was examined. Thus, to investigate whether GW501516 down-regulated ADAM10, T24 cells were stimulated with increasing concentrations of the PPARβ/δ agonist (0, 1, 15, 25 µM) and assessed for mRNA and protein expression by RTq-PCR and Western-blotting analyses. As shown in Figure 6A,B, ADAM10 expression was not modified by the drug at both mRNA and protein levels. And yet, GW501516 was active on gene expression since the *plin2* gene (perilipin 2), a known direct PPARβ/δ target [26], was increased after treatment from the concentration of 1 µM (Figure 6C). These findings suggested that the depletion of NTF upon GW501516 exposure was not the consequence of the decrease of ADAM10 expression but may be due to indirect action on Tspan15, a protein that regulates ADAM10 activity on N-cadherin.

### 3.7. GW501516 Exposure Reduced Tspan15 Expression in T24 Cells

**Figure 7:**
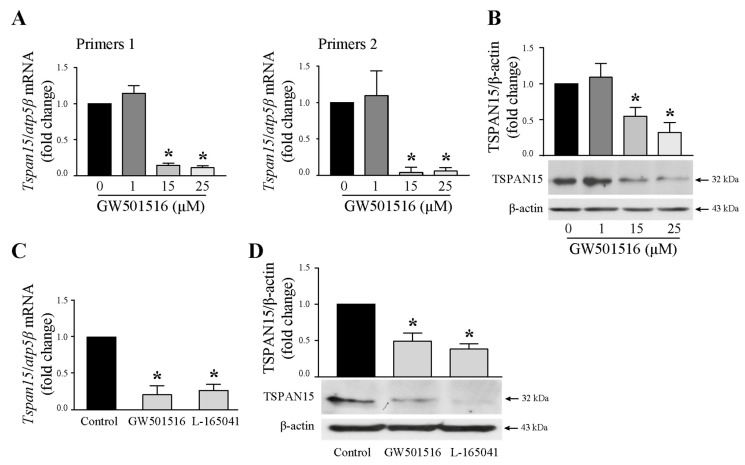
Tspan15 is down-regulated by GW501516 in T24 cells. Cells were treated with increasing concentrations of GW501516 (1, 15, 25 µM) for 24 h. (**A**) *Tspan15* mRNA expression was analyzed by RTq-PCR with two different primer pairs (primers 1 or primers 2). Fold inductions represent a comparison with vehicle-treated cells (set at 1) in the absence of GW501516. Data are means ± SD of three independent experiments performed in triplicates (* *p* < 0.05). (**B**) Western blotting analysis of TSPAN15 protein in control and stimulated cells. β-actin was used as an internal loading control. The graph depicts densitometric analysis results of Western blots by using ImageJ. Data are means ± SD of three independent experiments performed in triplicates (* *p* < 0.05). (**C**) Cells were stimulated by 15 µM GW501516 or 15 µM L-165041 (another PPARβ/δ agonist) for 24 h. *Tspan15* mRNA expression was analyzed by RTq-PCR. Fold inductions represent a comparison with vehicle-treated cells (set at 1) in the absence of agonists. Data are means ± SD of three independent experiments performed in triplicates (* *p* < 0.05). (**D**) Western blotting analysis of TSPAN15 protein in control and stimulated cells. β-actin was used as an internal loading control. The graph depicts densitometric analysis results of Western blots by using ImageJ. Data are means ± SD of three independent experiments performed in triplicates (* *p* < 0.05).

We have demonstrated that Tspan15 inhibition by RNA interference strategy led to a decrease of N-cadherin ectodomain shedding and thus to a diminution of NTF releasing. We showed also that GW501516 decreased the NTF amount in T24 cell-supernatants, but without targeting ADAM10 directly. Since Tspan15 is involved in ADAM10-mediated cleavage of N-cadherin, we then wondered whether GW501516 could act by regulating the expression of Tspan15. T24 cells were, therefore, treated with increasing concentrations of the drug (1, 15, 25 µM) for 24 h. Tspan15 mRNA level was evaluated by RT-qPCR with two different primer pairs. As shown in Figure 7A, there was a significant decrease in transcripts with primer 1 and primer 2 in a dose-dependent manner from 15 µM GW501516. This was correlated with the Tspan15 protein level as evidenced by Western blotting in total cell lysate (Figure 7B). The inhibitory effect of PPARβ/δ activation on Tspan15 expression was not specific to GW501516. It was also observed with another agonist, L-165041, at both mRNA (Figure 7C) and protein levels (Figure 7D) of Tspan15. It is important to note that GW501516 did not modify the level of the other TspanC8 mRNA (Figure S1).

### 3.8. GW501516 Acts through PPARβ/δ-Dependent Mechanisms on Tspan15 Expression in T24 Cells

**Figure 8:**
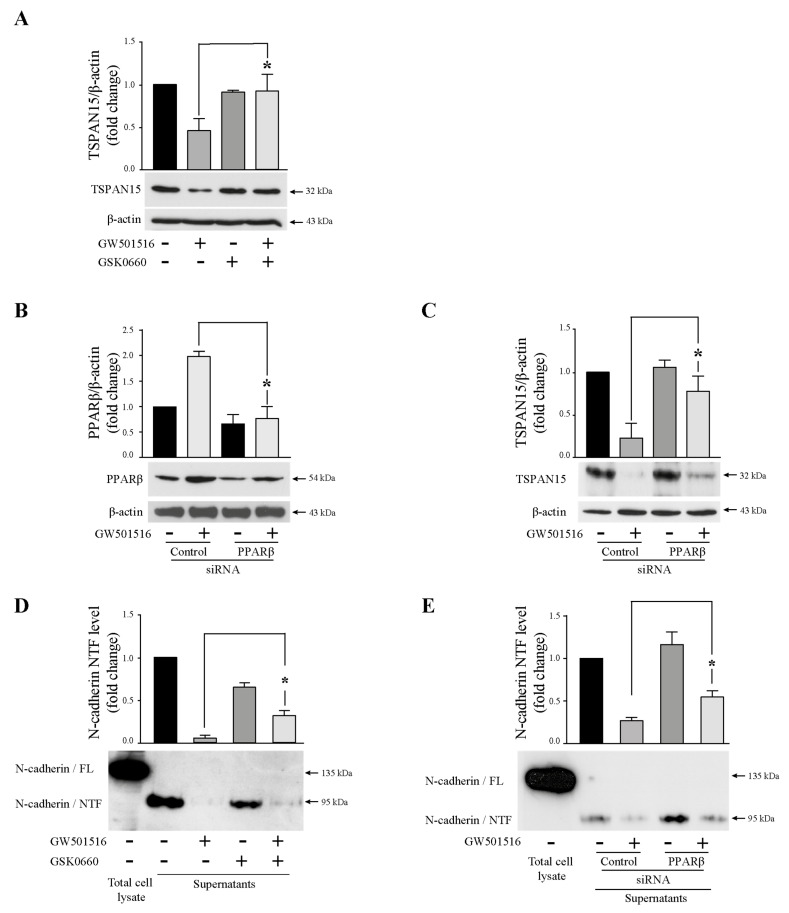
Tspan15 expression depends on PPARβ/δ transactivation. (**A**) T24 cells were treated with 15 µM GW501516 in the absence or presence of 10 µM GSK0660 (a PPARβ/δ antagonist) for 24 h. TSPAN15 protein expression was analyzed by Western blotting from control and stimulated cells. β-actin was used as an internal loading control. The graph depicts densitometric analysis results of Western blots by using ImageJ. Data are means ± SD of three independent experiments performed in triplicates (* *p* < 0.05). (**B**) Western blotting analysis of PPARβ/δ protein depletion in control and GW501516-stimulated cells transfected with PPARβ/δ siRNA or a control siRNA. β-actin was used as an internal loading control. The graph depicts densitometric analysis results of Western blots by using ImageJ. Data are means ± SD of three independent experiments performed in triplicates (* *p* < 0.05). (**C**) Western blotting analysis of TSPAN15 protein in control and GW501516-stimulated cells transfected with PPARβ/δ siRNA or a control siRNA. β-actin was used as an internal loading control. The graph depicts densitometric analysis results of Western blots by using ImageJ. Data are means ± SD of three independent experiments performed in triplicates (* *p* < 0.05). (**D**) N-cadherin/NTF level detection in T24 cell supernatants by Western blotting from cells stimulated with GW501516 alone or in combination with GSK0660. Total cell lysate was used as a positive control for N-cadherin expression. The graph depicts densitometric analysis results of Western blots by using ImageJ. Data are means ± SD of three independent experiments performed in triplicates (* *p* < 0.05). (**E**) N-cadherin/NTF level detection in T24 cell-supernatants by Western blotting from cells transfected with PPARβ/δ siRNA or a control siRNA. Total cell lysate was used as a positive control for N-cadherin expression. The graph depicts densitometric analysis results of Western blots by using ImageJ. Data are means ± SD of three independent experiments performed in triplicates (* *p* < 0.05).

To determine whether PPARβ/δ transactivation was essential for GW501516-promoted decrease of Tspan15 expression, T24 cells were treated by 25 µM drug alone or in combination with 10 µM GSK0660, a selective PPARβ/δ antagonist. Decreased Tspan15 was clearly observed when exposed to GW501516 alone but not when combined with GSK0660. The effect of GW501516 on the decrease of the Tspan15 protein level was significantly reversed by GSK0660 (Figure 8A), meaning that the molecule acts through PPARβ/δ activation since after the blockade of the receptor, the level of Tspan15 was no more diminished. An RNA interference method was then applied to knockdown PPARβ/δ expression. T24 cells were transfected with 50 nM PPARβ/δ siRNA or a control siRNA and then stimulated or not with 25 µM GW501516. In control siRNA-transfected cells, PPARβ/δ level was increased 2-fold in GW501516-treated cells compared to untreated cells. As expected, PPARβ/δ silencing was observed in T24 cells transfected with PPARβ/δ siRNA compared to cells transfected with a control siRNA in control cells (Figure 8B). In PPARβ/δ knockdown cells, the increase of the receptor was abolished upon GW501516 exposure (Figure 8B). As presented in Figure 8C, Tspan15 down-regulation by GW501516 was reversed by siRNA-mediated knockdown of PPARβ/δ. Consequently, the decrease of NTF production upon GW501516 exposure was reversed by the PPARβ/δ antagonist (Figure 8D) and the silencing of the nuclear receptor PPARβ/δ by a specific siRNA (Figure 8E).

Collectively, these results showed that GW501516 decreased Tspan15 expression and, as a result, the generation of N-cadherin/NTF through PPARβ/δ-dependent mechanisms. Both transactivation of the receptor and its presence in T24 cells are responsible for the effect of PPARβ/δ agonists.

## 4. Discussion

In view of its aberrant expression in carcinomas and especially in urothelial bladder cancer [6], its role in cell motility, invasion, angiogenesis, and resistance to apoptosis, N-cadherin is a prime target for anti-tumor therapies. Molecular targeting of this protein to inhibit its expression and/or block its function is a relevant approach to prevent tumor progression and the development of metastases. As such, several antagonists have been developed: monoclonal antibodies, and linear or cyclic synthetic peptides, targeting the EC1 to EC4 motifs of the extracellular domain, thus blocking the intercellular adhesion and interactions of N-cadherin with the FGFR pathway [27]. However, these anti-cancer molecules are not currently used to treat patients. However, they are still undergoing clinical trials. It should be noted that they have no effect on the N-cadherin cleavage process. They are potentially able to block the function of the full-length N-cadherin. Since N-cadherin is cleaved by ADAM10, these N-cadherin antagonists are also able to inhibit the function of NTF. However, they do not affect the release of CTF2, which can act as a proliferating gene transcription regulator.

NTF generation results from ADAM10 proteolytic activity on N-cadherin ectodomain [17]. Elevated levels of this pro-angiogenic peptide have been detected in the serum of cancer patients [14]. In this context, our study focused on the effect of a synthetic PPARβ/δ agonist, GW501516, with the aim of blocking NTF production by acting on N-cadherin cleavage. GW501516 is reported to inhibit pancreatic cancer cell invasion through the suppression of MMP-9 expression [28]. It blocked breast cancer cell migration and invasion [29]. It inhibited tumor growth in mice xenografted with MDA-MB-231 cells through PPARβ/δ/c-MYC interaction [30]. It triggered nasopharyngeal carcinoma cell apoptosis [31] and decreased hepatoma cell proliferation [32]. In previous work, we demonstrated that exposure of proliferating T24 invasive bladder cancer cells to 25 µM GW501516 led to a diminution in cell viability, a G2/M cell cycle arrest, and the triggering of apoptosis [22]. In addition, in the same cells, 15 µM of the drug decreased the full-length N-cadherin level [23]. In this study, we confirmed our results on the full-length N-cadherin since we showed that GW501516 decreases *cdh2* expression; it diminishes the N-cadherin whole form level, and we demonstrated for the first time in T24 invasive bladder cancer cells, that it does not modify ADAM10 expression but decreases the expression of Tspan15 (mRNA and protein), a regulator of ADAM10. All this tends to reduce the NTF level in the extracellular environment.

We have highlighted a decrease in *cdh2* gene expression, which could result from regulation at the transcriptional level through a PPARβ/δ-dependent mechanism. In general, the genomic action of PPARβ/δ involves its binding, as a heterodimer with RXR, to a PPRE sequence, then its activation by an agonist, leading to the release of corepressors and the recruitment of co-activators. However, no PPRE sequence has been identified in the promoter of *cdh2*. GW501516 could then increase the expression of a corepressor, or decrease that of a co-activator, or block its transcriptional activity. BCL-6 is a PPARβ/δ-associated transcriptional repressor [28] and GW501516-activated PPARβ/δ releases this repressor, which suppresses the function of NFκB, a *cdh2* activator binding to a NFκB site located in the *cdh2* promoter region [33]. A microRNA could also be responsible for the decrease in *cdh2* expression. Interestingly, miR-124 targets the *cdh2* transcript [34], and above all it has a promoter containing a PPRE sequence [35]. Therefore, GW501516-activated PPARβ/δ could increase the expression of miR-124, which would induce the degradation of *cdh2* transcripts and/or the inhibition of their translation, which would also explain the decrease in the protein level of N-cadherin. Further investigations are needed to validate all these hypotheses.

ADAM10 is overexpressed in many cancers and linked to tumor progression. Its implication in urothelial carcinogenesis is poorly documented. ADAM10 is highly expressed in bladder cancer tissues compared to normal bladder counterparts and it is associated with elevated tumor stage and grade. ADAM10 knockdown leads to decreased cell proliferation, migration, and invasion and induces chemoresistance of bladder cancer cells [15]. In this context, ADAM10 has emerged as a promising therapeutic goal to treat cancer. But, to date, no inhibitory therapy directed against ADAM10 has been approved. This is because the activity of this enzyme is beneficial in other pathologies such as Alzheimer’s disease [36]. Considering the positive and negative effects of ADAM10 depending on the pathology under consideration, and thus to avoid deleterious side effects, targeting ADAM10 activity specifically to a particular substrate is more relevant.

ADAM10 is a TspanC8-associated protein. Each of the six described TspanC8-ADAM10 proteolytic complexes is characterized by a substrate selectivity based on different conformations of ADAM10 in each complex. TspanC8 is involved in the exit of ADAM10 from the endoplasmic reticulum, its maturation, and its localization in late endosomes or at the cell surface [37,38]. The Tspan15/ADAM10 was identified to mainly cleave N-cadherin. In addition, this TspanC8 interacts with ADAM10 in the endoplasmic reticulum (ER), causing its maturation and promoting its transport to the plasma membrane by speeding up its exit from ER and stabilizing its active form [17,18,19,39]. Our data demonstrated, for the first time in T24 invasive bladder cancer cells, the requirement of Tspan15 in ADAM10-mediated shedding of N-cadherin ectodomain. More interestingly, we show for the first time that while GW501516 had no effect on ADAM10, it reduced the expression of Tspan15 at both mRNA and protein levels. To date, no study has described the structure of the *Tspan15* gene promoter, so no PPRE sequence has been identified. Nevertheless, GW501516 acted through PPARβ/δ-dependent mechanisms since in combination with an antagonist or in the presence of PPARβ/δ-siRNA, the decreased expression of Tspan15 was reversed, and, consequently, the diminution in soluble N-cadherin was much less important. The Tspan15-decrease induced by the PPARβ/δ agonist could be due to the overexpression of a transcriptional repressor, the inhibition of an activator, or the action of a miRNA. At present, none of these mechanisms have been mentioned in the scientific literature. However, one study reported the inhibitory effect of miR-339-5p on Tspan15 expression [40]. GW501516 could then increase the expression of this miRNA.

The therapeutic modulation of Tspan15 expression is an attractive approach to treating cancer since Tspan15 is overexpressed in several cancers. One study reported that the expression of Tspan15 (mRNA and protein) was increased in squamous cell carcinomas of the esophagus (OSCC) and in OSCC-derived cell lines. Moreover, the invalidation of the *Tspan15* gene inhibits the migratory and invasive capacities of the cells without affecting their proliferation. Thus, Tspan15 may activate signaling pathways that cause metastasis formation. In addition, ADAM10 expression was reduced in Tspan15-depleted cells. This was associated with less production of N-cadherin/NTF and weak immunodetection of nuclear β-catenin. The Tspan15-mediated pathway for OSCC formation would involve ADAM10, N-cadherin, and β-catenin [41]. Furthermore, positive immunodetection of Tspan15 in hepatocellular carcinoma was correlated to cancer recurrence, and its overexpression in hepatoma cell line HepG2 increased cell proliferation through CTGF (connective tissue growth factor) production and ERK1/2 phosphorylation [42]. To date, no data are available on the expression of Tspan15 in relation to the grade and stage of bladder cancer.

In our T24 bladder cancer cell model, after treatment of cells with GW501516, a decrease in the soluble N-cadherin level was detected in the extracellular compartment, suggesting a reduction in proteolytic cleavage of N-cadherin, possibly due to a decrease in the presence of Tspan15 at the plasma membrane. We detected Tspan15 in T24 cells and demonstrated that it was crucial for ADAM10-mediated cleavage of N-cadherin. Importantly, we showed that GW501516 inhibited only Tspan15 without affecting ADAM10 in these cells. Thus, Tspan15 would no longer interact with ADAM10 and therefore would not allow the metalloproteinase to cleave N-cadherin. The GW501516-mediated decrease in Tspan15 expression would have two consequences: it reduces Tspan15/ADAM10 interaction thus impacting ADAM10 maturation and membrane addressing and therefore its activity; both effects contribute to the inhibition of N-cadherin cleavage. Other experiments are needed and in progress to clarify this point.

## 5. Conclusions

Our results reveal the potential therapeutic interest of GW501516 and thus of PPARβ/δ agonists. This drug reduces N-cadherin cleavage by acting directly on Tspan15. It reduces its expression, thus impacting ADAM10 activity on N-cadherin cleavage without affecting the expression of ADAM10. This could have several advantages: blocking the role of Tspan15 in the development of metastases, reducing the production of NTF and CTF-2 fragments and thus their functions (activation of angiogenesis for NTF and proliferative effect for CTF-2), and preventing the release of β-catenin and its transcriptional action on proliferative genes. GW501516 could potentiate the effect of N-cadherin antagonists such as ADH-1 or GC-4, which target the ectodomain of the protein. Such a combined treatment would allow GW501516 to maintain the full-length N-cadherin, while ADH-1 (or GC-4) would block the action of N-cadherin. This therapeutic combination could be more effective than N-cadherin antagonists alone in the treatment of cancer, particularly bladder cancer.

## Data Availability

The data presented in this study are available in this article.

## Supplementary Materials

The following supporting information can be downloaded at: https://www.mdpi.com/article/10.3390/cells13080708/s1. Table S1: Primer and probe sequences used in RTq-PCR. Figure S1: TspanC8 mRNA expression in GW501516-treated T24 cells. Figure S2: GW501516 decreases the viability of T24 cells.
