# Supplementary material for: GW501516-Mediated Targeting of Tetraspanin 15 Regulates ADAM10-Dependent N-Cadherin Cleavage in Invasive Bladder Cancer Cells
Source: Cells. 2024 Apr 19;13(8):708. doi: 10.3390/cells13080708 (PMC11049359; doi:10.3390/cells13080708)
Supplement: Supplementary file 1 [file cells-13-00708-s001.zip › Cells 2929450 Supplementary data V3 S. FAUCONNET.pdf]

Supplementary Materials and Methods:

Cell viability assay

Cell viability of T24 cells was determined by the Cell Proliferation Kit 1 (MTT) (Roche Diagnostics, Meylan, France) according to the manufacturer's guidelines. 20,000 cells/well were seeded in 96-well plates. Cells were stimulated in serum-free medium with or without (control cells) different concentrations of GW501516 (1, 5, 10, 15, 20, 25 µM) for 24 h. MTT labeling reagent was then added for 4 h at 37°C leading to the formation of insoluble formazan crystals. After the addition of a solubilization solution (100 µL/well), the formazan dye was quantified by spectrophotometry (ELISA reader, parkin Elmer). The measured absorbance [A572 nm – A635 nm] directly correlated with the number of viable cells.

Supplementary data

Figure legends

Figure S1. TspanC8 mRNA expression in GW501516-treated T24 cells. Cells were treated with increasing concentrations of GW501516 (1, 15, 25 µM) for 24 h. *TspanC8* mRNA expression was analyzed by RTqPCR with specific primer pairs for *Tspan5* (**A**), *Tspan14* (**B**), *Tspan17* (**C**), and *Tspan33* (**D**). Fold inductions represent comparison with vehicle-treated cells (set at 1) in the absence of GW501516. Data are means ± SD of three independent experiments performed in triplicates.

Figure S2. GW501516 decreases the viability of T24 cells. Cells were incubated with different concentrations of GW501516 (1, 5, 10, 15, 20, 25 µM). The cytotoxic effect of the PPARβ/δ agonist was assessed by MTT assay. Data are means ± SD of three independent experiments performed in triplicates (* $p < 0.05$).
