# Supplementary material for: GW501516-Mediated Targeting of Tetraspanin 15 Regulates ADAM10-Dependent N-Cadherin Cleavage in Invasive Bladder Cancer Cells
Source: Cells. 2024 Apr 19;13(8):708. doi: 10.3390/cells13080708 (PMC11049359; doi:10.3390/cells13080708)
Supplement: Supplementary file 1 [file cells-13-00708-s001.zip › Cells 2929540 Table S1 V3 S.FAUCONNET.pdf]

Table S1

Primer and probe sequences used in RTqPCR.

Target	Primers (5' → 3')		Probe
Cdh2	For	tgggaatccgacgaatgg	6-FAM-tgaaagacccatccacgctgagcc-BHQ-1
	Rev	gcagatcggaccggatactg	
ATP5β	For	tactgtcgcgtgccattgct	6-FAM-atcccaacattgttggcagt-BHQ-1
	Rev	cacgggcaacatcgtaatgc	
Adam10	For	aaacaccagcgtgccaaaag	
	Rev	ccctcttcattcgtaggttgaaa	
Plin2	For	tgagatggcagagaacggtgtgaa	
	Rev	ttgcggctctagcttctggatgat	
Rpl38	For	gttgctgcttgctgtgagtg	
	Rev	cgaatttggcatcctttcgtc	
	For	acaagggtcctgaagtcagtt	
Tspan5	Rev	tgatggaagagctgttggacaga	
	For	ctgcgtcaagtatctgatcttcc	
Tspan10	Rev	aagccacgtaacagggagg	
	For	ggctctgcgggagaatatctg	
Tspan14	Rev	gcactggttagctttctgaagg	
	For	acttcctgaacgacaacattcg	
Tspan15	Rev	cgccacagcactgaacttgattt	
	For	ctgctgcgggaaatacttcct	
Tspan17	Rev	gatgttcgagagaacgcccctt	
Tspan33	For	ctacgatcggctaatgaagca	
	Rev	tgagcaggaacatgaggacac	
